# Expression pattern of Protein Kinase C ϵ during mouse embryogenesis

**DOI:** 10.1186/1471-213X-13-16

**Published:** 2013-05-02

**Authors:** Sergio Carracedo, Ursula Braun, Michael Leitges

**Affiliations:** 1 Biotechnology Centre of Oslo, University of Oslo, Gaustadalleen 21, Oslo, N-0349, Norway

**Keywords:** Novel protein kinase C, PKC epsilon, Mouse embryogenesis, Lac Z, Ganglia

## Abstract

**Background:**

Protein kinase C epsilon (PKCϵ) belongs to the novel PKC subfamily, which consists of diacylglycerol dependent- and calcium independent-PKCs. Previous studies have shown that PKCϵ is important in different contexts, such as wound healing or cancer. In this study, we contribute to expand the knowledge on PKCϵ by reporting its expression pattern during murine midgestation using the LacZ reporter gene and immunostaining procedures.

**Results:**

Sites showing highest PKCϵ expression were heart at ealier stages, and ganglia in older embryos. Other stained domains included somites, bone, stomach, kidney, and blood vessels.

**Conclusions:**

The seemingly strong expression of PKCϵ in heart and ganglia shown in this study suggests a important role of this isoform in the vascular and nervous systems during mouse development. However, functional redundancy with other PKCs during midgestation within these domains and others reported here possibly exists since PKCϵ deficient mice do not display obvious embryonic developmental defects.

## Background

The mammalian protein kinase C family embraces ten serine/threonine kinase isoforms divided into three subfamilies: classical PKCs (cPKCs, α, β_Ι_, β_ΙΙ_ and γ), novel PKCs (nPKCs, δ, ϵ, η and θ), and atypical PKCs (aPKCs, ζ , ι/λ). Like all nPKCs, PKCϵ does not depend on calcium but on diacylglycerol to become active, since its C2 domain-like sequence lacks residues with calcium-coordinating side chains [1]. Regarding PKCϵ regulation, phosphorylation of the PKCϵ catalytic domain is required so that PKCϵ can reach full enzymatic activity [2], whereas phosphorylation of one of the serine residues of its regulatory domain enables the interaction of PKCϵ with 14-3-3 proteins [3]. In addition to the regulatory domains of the PKC family, PKCϵ also contains an exclusive motif that binds actin and allows for its phosphorylation [4]. Therefore, actin, as well as other proteins, such as histone1 [5] or TRAM [6], can interact and serve as PKCϵ substrates. In addition, phosphorylation by PKCϵ of molecules such as Akt [7] or PKD [8] make evident the significant role of this nPKC isoform in the regulation of cell signaling. PKCϵ also regulates ion channels, such as GABA_A_ receptors [9], or cytoskeleton proteins, as for example vimentin [10] and actin as above mentioned [4,11]. PKCϵ is also involved in different cellular events, as for example cell migration [12] and cell division [13]. Although PKCϵ deficient mice are viable [14], they display several phenotypes, such as impaired cutaneous wound closure [15], improved glucose-induced insulin secretion and reduction of insulin clearance [16], and glomerulosclerosis and tubulointerstitial fibrosis [17]. In addition, PKCϵ has been suggested as a promoter of different types of cancer [18-20]. Hence, PKCϵ also appears important in pathological scenarios. However, the overall expression pattern of this isoform has not been reported yet. In this study, we show the spatiotemporal expression of PKCϵ in mouse during embryogenesis by using PKCϵ null embryos containing the LacZ reporter gene under the control of the endogenous PKCϵ promoter [14], and by staining wild type embryos with antibodies to PKCϵ. Thus, these data contribute to the general understanding of the expression of murine PKCϵ *in vivo*, and to ellucidate exclusive or redundant roles of PKCϵ.

## Results and discussion

### PKCϵ expression at E8.5 and E9.5

**Figure 1:**
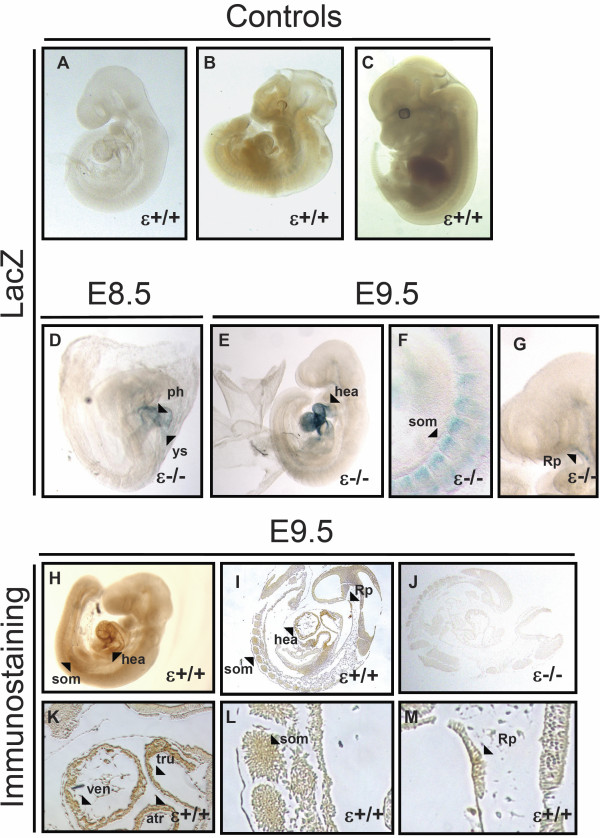
**PKCϵ expression at E8.5 and E9.5. A-C**, lack of Lac Z signal due to endogenous β-galactosidase was confirmed on wild type embryos (controls). **D**, at E8.5, X-Gal staining is detected in the yolk sac and primitive heart (ys and ph, respectively). **E-G**, at E9.5, developing heart (hea), somites (som) and Rathke’s pouch (Rp) showed LacZ signal. **H-M**, Antibody staining of 5 μm E.9.5 embryo sections confirmed the expression of PKCϵ in somites, Rathke’s pouch and heart. In the latter domain, immunoreactivity was detected at the walls of the primitive ventricle (ven), atrium (atr) and truncus arteriosus (tru). E9.5 PKCϵ deficient embryo sections were used as a negative control for the antibody (**J**).

PKCϵ analysis through LacZ staining was performed from E8.5 to E14.5. Given the absence of obvious developmental phenotypes, homozygous embryos were chosen to report LacZ stained domains due to their increased signal, mainly at earlier stages, versus their heterozygous littermates. In addition, antibody staining with corresponding negative controls was performed at E9.5 and E13.5 as a control for the specifity of the LacZ signal. Moreover, to confirm the absence of endogenous β-galactosidase activity in our stainings, wild type littermates underwent the same LacZ staining protocol (Figure 1A-C). Background problems using *in situ* hybridization prevented us from obtaining reliable signal, and therefore data obtained with this technique were not included here. However, there is a recent *in situ* hybridization study of the mouse transcriptome at E14.5 that shows mRNA expression of PKCϵ at domains that we also report here [21,22], which further strengthens our data.

At E8.5, Lac Z activity was mainly detected at the primitive heart, along with some weak signal in the yolk sac (Figure 1D). LacZ signal was noticeably increased at E9.5 in the developing heart (Figure 1E), and weak novel staining was detected at somites and Rathke’s pouch (Figure 1F and G respectively). Consistently, immunostaining of WT whole embryos and sections of the embryonic stage showed similar staining pattern, with heart, somites and Rathke’s pouch as the domains showing immunoreactivity (Figure 1H and I). PKCϵ deficient embryo sections were used as a negative control (Figure 1J). A closer look at the heart on immunostained sections allowed us to observe PKCϵ expression mainly at the walls of the primitive ventricle (ven), atrium (atr) and truncus arteriosus (tru) (Figure 1K). Immunodetection of PKCϵ at this stage could be detected at most cells in somites (Figure 1L) and Rathke’s pouch (Figure 1M). The absence of signal in PKCϵ deficient sections and the overlapping pattern obtained through both staining methods confirmed the specifity of the signal at the reported domains through both approaches.

### PKCϵ expression from E10.5 to E12.5

**Figure 2:**
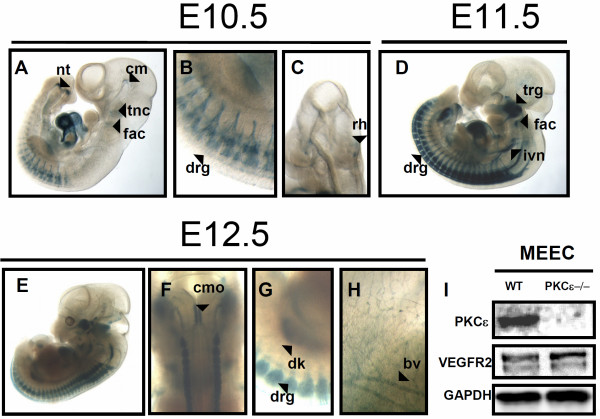
**PKCϵ expression in whole mount embryos from E10.5 to E12.5. A -C**, At E10.5, trigeminal neural crest (tnc), facio-acoustic neural crest complex (fac), neural tube in caudal region of the tail (nt), cephalic mesenchyme (cm), dorsal root ganglia (drg), and roof of the hindbrain (rh) show reporter activity. **D**, At stage E11.5, staining of the trigeminal (V) ganglion (trg), facio-acoustic (VII-VIII) ganglion complex (fac), dorsal root ganglia (drg), as well as interior ganglion of vagus (X) nerve (ivn) became all prominently LacZ stained. **E-H**, At E12.5, novel LacZ activity in the caudal part of the medula oblongata (cmo) and developing kidney (dk) can be detected. Embryos at this stage also show clear LacZ signal in blood vessels (fig. H), which was weakly detectable from E9.5. **I**, Western blotting showing expression of PKCϵ in wild type mouse embryonic endothelial cells (MEECs). PKCϵ deficient MEEC were used as negative control and VEGFR2 was used as endothelial marker.

In E10.5 whole mount embryos, the nervous system became noticeable through β-galactosidase staining. Thus, neural tube in caudal region of tail, dorsal root ganglia, trigeminal (V) neural crest, facio-acoustic (VII-VIII) neural crest complex all showed Lac Z signal, along with cephalic mesenchyme tissue and roof of the hindbrain (Figure 2A-C). X-Gal staining was still strong in the heart at this stage and onwards.

At E11.5, dorsal root ganglia and trigeminal (V) ganglion was strongly stained. Moreover, fascio-acustic (VII-VIII) ganglion complex, and interior ganglion of vagus (X) nerve all showed prominent LacZ reporter gene expression (Figure 2D). The strong LacZ signal observed in heart and ganglia at this stage and onwards suggests an important role of PKCϵ within the vascular and nervous systems. However, the lack of obvious developmental defects suggests the existence of functional redundancy with other PKC isoforms. PKCδ might be a candidate in this regard given its similar expression pattern [23].

At E12.5, whole mount lacZ staining still showed clear signal in the neural and cardiovascular systems (Figure 2E-H), but in addition signal at the caudal part of medula oblongata (Figure 2F) and kidney (Figure 2G) could be observed. In addition, although LacZ signal in blood vessels was first weakly detected from E9.5, it was from E12.5 when PKCϵ expression in this domain was very obvious (Figure 2H). However, PKCϵ in blood vessels could not be detected via immunostaining even at later stages, when the LacZ activity in endothelium was even clearer. Since PKCϵ appears expressed *in vitro* in mouse embryonic endothelial cells (Figure 2I and Additonal file 1: Figure S1) as well as in endothelial cells from other species [24-26], we speculate that endothelial PKCϵ expression *in vivo* is too low to be detected in mouse by the antibody and/or protocol we used in our immunohistochemistry. Hence, detection of PKCϵ expression in endothelium via LacZ staining was perhaps possible because the Neo cassette that was used to generate PKCϵ deficient mice [15] upregulated PKCϵ expression in endothelium, as previously shown for other genes and tissues [27].

### PKCϵ expression at E13.5 and E14.5

**Figure 3:**
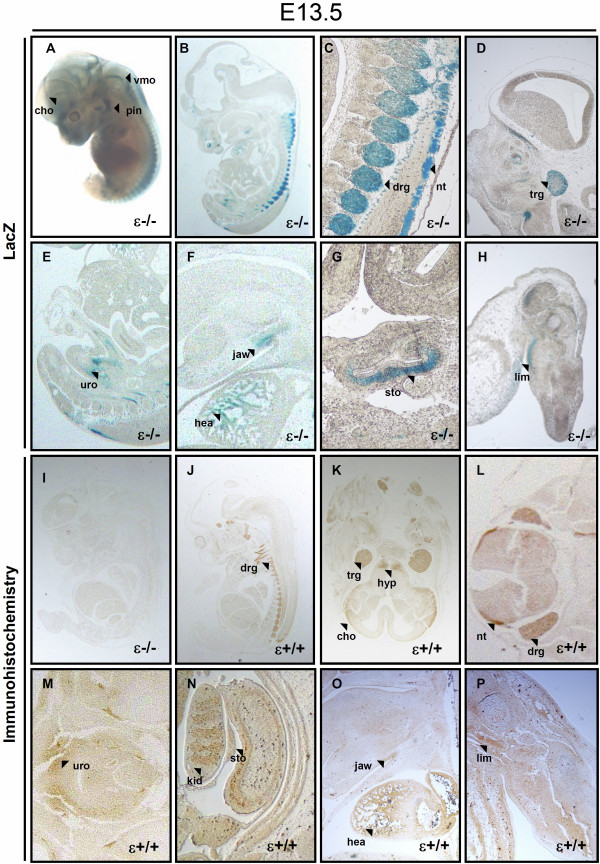
**PKCϵ expression is highest within the nervous system at E13.5. A**, at embryonic stage E13.5, whole mount staining allowed for the detection of LacZ signal in choroid plexus (cho), ventral part of the medulla oblongata (vmo) and pinna of the ear (pin). **B-H**, 15 μm sagittal sections at this stage showed incomplete penetration of X-Gal at previously reported domains such as dorsal root ganglia (drg) and trigeminal (V) ganglion (trg). Lac Z signal at this stage was also detected in neural tube (nt), urogenital sinus (uro), caudal part of lower jaw (jaw), heart (hea), mucosal lining of the stomach (sto), and limbs (lim). **I**, Immunohistochemistry at E13.5 included staining of PKCϵ deficient sections as negative control. **J**, sagittal sections confirmed ganglia as the domain with highest expression of PKCϵ at this stage. **K-P**, Immunostaining of cross sections (**K**-**N**) and sagittal sections (**O** and **P**) confirmed PKCϵ detected via LacZ staining in trigeminal ganglion (trg), choroid plexus (cho), neural tube (nt), urogenital sinus (uro), mucosal lining of the stomach (sto), kidney (kid), heart, caudal part of lower jaw (jaw) and limbs (lim). In addition, it also allowed for detection of PKCϵ in hypothalamus (**K**, hyp).

In addition to blood vessels, ganglia, and other LacZ-stained domains described at previous stages, reporter activity at E13.5 was detected in the pinna of the ear, chroroid plexus and ventral part of medulla oblongata (Figure 3A). A closer look on E13.5 LacZ-stained sections showed that only some and not all cells in the trigeminal and dorsal root ganglia were responsible for the strong X-Gal staining observed on these domains (Figure 3B-D). This might be due to penetration related issues of X-Gal, which would have prevented the staining of the whole domain. β-galactosidase activity was also observed in urogenital sinus, dorsal part of the jaw, heart, mucosal lining of the stomach and limbs (Figure 3E-H). The signal detected at the heart was located at the trabeculated part of the wall of the ventricles (Figure 3F). Immunostaining of sagittal and cross sections at E13.5 could confirm PKCϵ expression at domains previously reported via LacZ staining as well as other new domains that were not detected through LacZ activity. However, such domains were not stained in PKCϵ deficient mouse sections (Figure 3I) and therefore appear specific. Ganglia was the most prominently immunostained domain, as observed via LacZ. Thus, antibodies allowed for detection of PKCϵ at dorsal root ganglia (Figure 3J and L), hypothalamus, trigeminus and cerebral cortex (Figure 3K), neural tube (Figure 3L), urogenital sinus (Figure 3M), kidney and mucosal lining of the stomach (Figure 3N), heart (Figure 3O) and limbs (Figure 3P).

**Figure 4:**
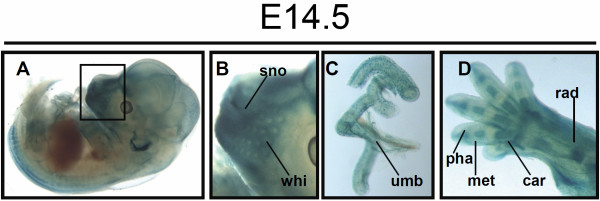
**PKCϵ expression at E14.5. A **and its inset magnified in **B **show LacZ signal in the snout area (sno) except for whiskers (whi). **C**, β-Gal activity was detected in umbilical vein (umb). **D**, precartilage of primordium of radius (rad) phalangeal (pha), metacarpal (met) and carpal (car) bones (fig. V) all became clearly LacZ stained.

As expected, and consistent with previous mRNA analysis of embryos at E14.5 [22], PKCϵ was still detected at domains such as root ganglia or brain (Figure 4A). However, at this stage X-Gal staining was also observed on yet unreported domains at the mRNA level, such as blood vessels (already detected in this study at earlier stages), snout –except for whiskers (Figure 4B), umbilical chord (Figure 4C), and precartilage primordium of bone, mainly at forelimbs and hindlimbs. Thus, radius, precartilage primordium of phalangeal, metacarpal and carpal bone were showing remarkable LacZ activity (Figure 4D).

## Conclusions

Our expression pattern for PKCϵ during mouse midgestation suggests that at least several domains, such as ganglia, vascular system, cartilage primordium or stomach, express this novel PKC isoform. However, heart and nervous system appear as the main sites of expression for PKCϵ. More specifically, dorsal root ganglia and trigeminal (V) ganglia are the domains where PKCϵ seems to be most prominently expressed. Thus, as already indicated by mouse *ex vivo* studies [28] and *in vivo* work performed in rats [29], these data suggest that PKCϵ likely has an important *in vivo* role within the nervous system in mice. The fact that there is no reported phenotype or functional deficiency in the nervous system in mice suggests the existence of functional redundancy among members of the PKC family. Indeed, data regarding transcription of PKC δ [30] and ϵ [21] within the same domains of the nervous system already exist as part of a study that uses an *in situ* hybridization approach to show the expression pattern of a high number of transcripts in the mouse embryo [22]. In addition, our recent description of the expression pattern for PKC δ at the protein level during mouse midgestation [23] together with the present study also show the co-existence of these two nPKC isorforms within the vascular and nervous systems. Thus, the expression pattern of PKCϵ may contribute to address such redundancy by pointing us towards domain(s) causing potential lethality in mice lacking several PKC isoforms.

## Methods

### Animals and embryo collection

Mice deficient for PKCϵ have been previously described [14]. All the animal work was approved by the Folkehelse Institute in Oslo (Norway), and performed according to its institutional guidelines and rules and regulations of the Federation of European Laboratory Animal Science Association’s (FELASA). Pregnancy stages were assigned upon observation of vaginal plug at approximately midday, which was considered as E0.5.

### Cell culture

Wild type and PKCϵ deficient mouse embryonic endothelial cells (MEECs) were generated by digesting E11.5 embryos in a collagenase/dispase cokctail (Roche) mixed with DMEM + pyruvate (Gibco) for 20 min at 37°C. Embryos were subsequently washed in PBS and incubated overnight in DMEM supplemented with pyruvate, 10 μg/ml endothelial cell growh supplement (Sigma) and 10% fetal calf serum (complete medium). The next day, medium containing debris and dead cells was removed, and supernatant obtained from cultured cells producing polyoma virus T large antigen (kindly donated by Urban Deutsch) was added together with 8 μg/ml polybrene (Sigma) for two hours in order to selectively immortalize endothelial cells [31]. Upon removing supernatant, complete medium was added and cells were left in culture for several weeks. As growing endothelial island appeared, removal of non-endothelial cells was manually performed with a pippet to allow endothelial islands to expand and reach confluence. To confirm the endothelial phenotype and the purity of our cells, VEGFR2 (Cell Signalling Technologies) and VE-Cadherin antibodies (AH diagnostics) were used to detect the corresponding endothelial markers via western blot (Figure 1I) and immunofluorescence microscopy (Additional file 1: Figure S1), respectively.

### Western blotting

Cells were washed in PBS, trypsinized, and the resulting pellets further lysed in SDS-sample buffer, sonicated, boiled for 3 min, and subjected to a SDS-PAGE on 8% gels. Proteins were then transferred onto nitrocellulose membranes (GE Healthcare). Membranes were blocked for 1 h at room temperature with 5% non-fat dry milk (Marvel) in PBS containing 0.05% Tween 20 (PBS-T), incubated with either primary antibody to VEGFR2 (Cell Signaling, 1:1000), PKCϵ (Santa Cruz biotechnologies, 1:1000) or GAPDH (Cell Signaling 1:5000) overnight at +4°C. Upon washing in PBS-T three times, membranes were further incubated with goat anti-rabbit horseradish peroxidase-conjugated secondary IgGs (Jackson Immunoresearch, 1:5000) for 3h at room temperature. Membranes were was three times and developed using the SuperSignal West Pico kit (Thermo-Scientific Pierce) and photographed using the ChemiDoc XRS device and the Quantity One 1-D analysis software (Bio-Rad).

### LacZ staining

Steps corresponding to fixation (4% paraformaldedyde in PBS) and washing/permeabilization (Na_2_HPO_4_ 85 mM, NaH_2_PO_4_ 16 mM, MgCl_2_ 2 mM, 0.01% Na-desoxycholate, 0.02% NP-40) were performed for either 5 min (embryos up to 9.5 dpc) or 15 min (embryos from 10.5 dpc) at room temperature. Upon isolation, embryos were fixed, washed three times, and incubated with gentle shaking and protected from light overnight at 37°C in staining solution (for 10 ml, 9.7 ml of washing solution, 200 μl of K_3_ [Fe(CN)_6_] 0.5 M, 200 μl of K_4_[Fe(CN)_6_] 0.5 M, and 175 μl of 50 mg/ml X-Gal (Sigma-Aldrich) in DMSO were used). Next day, embryos were washed three times at room temperature and postfixed in 4% formalin in washing solution overnight at +4°C. PKCϵ deficient embryos (unless otherwise stated) were then passed into increasing concentrations of glycerol (25%, 50% and 80%) and photographed by using a Zeiss stereoscope equipped with camera and Axiovision software. LacZ stained embryos to be sectioned were instead postfixed in bouin’s solution (Sigma) the next day after β-gal staining was obtained, washed 3 times in PBS, passed into increasing concentrations of ethanol (30%, 50%, 70% and 100%, 2 washes per concentration), placed into a mix 1:1 of Ethanol-xylene, washed 2 times in xylene, and finally embedded in paraffin. In order to better observed LacZ domains, sections were obtained at a thickness of 15 μm and left overnight at room temperature. They were next subjected to dewaxing as follows: 2 washes in xylol for 10 min, 2 washes in absolute ethanol for 5 min, 1 wash in 70% ethanol for 2 min, and at least 5 min in distilled water. Sections were then mounted on mowiol (Polysciences) and photographed.

### Immunostaining

Upon fixation of embryos in 4% PFA overnight, embryos were washed 3 times in PBS, passed into increasing concentrations of ethanol (30%, 50%, 70% and 100%, 2 washes per concentration), placed into a mix 1:1 of Ethanol-xylene, washed 2 times in xylene, and finally embedded in paraffin. Five μm paraffin embedded sections of 9.5 and 13.5 dpc embryos were subjected to dewaxing as follows: 2 washes in xylol for 10 min, 2 washes in absolute ethanol for 5 min, 1 wash in 70% ethanol for 2 min, and at least 5 min in distilled water. Sections were subsequently boiled for two minutes in citric acid pH 6.0 for antigen retrieval, washed three times in PBS, bleached for 20 min with a mix of 30% peroxide, 1M HCl, and methanol with the ratio 1:1:100, respectively, rinsed in PBS, and incubated at 4°C overnight in rabbit polyclonal anti mouse PKCϵ (Santa Cruz Biotechnology) in a 1:200 dilution in PBS containing 5% fetal calf serum (FCS). Next day, sections were washed in PBS and incubated 4°C overnight in goat antirabbit IgGs conjugated to horse radish peroxidase (HRP, Jackson Immunoresearch) in a 1:200 dilution in PBS containing 5% FCS. Detection of PKCϵ was then analyzed using the DBA method according to the manufacturer’s instructions (Biogenex), rinsed in PBS and mounted in mowiol (Polysciences). For immunofluorescent detection of VE-Cadherin in MEECs (Additional file 1: Figure S1), cells were seeded in a 12 well-plate for two days, fixed in 4% PFA for 10 min at room temperature, rinsed three times in PBS for 5 min, permeabilized for 15 min with 10% goat serum in PBS 0.1% Triton X-100, blocked in PBS 10% sheep serum for 45 min, incubated in 1:200 rat anti mouse VE-Cadherin (AH Diagnostics) in PBS overnight at 4°C, rinsed three times in PBS, and incubated in 1:400 goat anti-rat cy3 (Jackson Immunoresearch) in PBS for three hours. Upon rinsing, immunofluorescence detection was performed using a Nikon Eclipse Ti microscope.

## Abbreviations

Dpc: Days post coitum; nPKC: Novel Protein Kinase C; PKCϵ: Protein kinase C epsilon; PKCδ: Protein kinase C delta.

## Competing interests

The authors declare no competing interests.

## Authors’ contributions

SC acquired, analysed and interpreted the data, and ellaborated the manuscript. ML and UB generated PKCϵ deficient mice. ML participated in the design and interpretation of the experiments, and helped to write the manuscript. All authors read and approved the final version of the manuscript.

## Supplementary Material

Additional file 1: Figure S1 Purity of MEEC lines. VE-Cadherin was detected via immunofluorescence microscopy to confirm the purity of WT and PKCϵ deficient MEECs. Nearly all cells appeared positively stained in both genotypes. WT mouse embryonic fibroblasts (MEFs) were used as a negative control. Click here for file
