# Supplementary figures and images for: Expression pattern of Protein Kinase C ϵ during mouse embryogenesis
Source: BMC Dev Biol. 2013 May 2;13:16. doi: 10.1186/1471-213X-13-16 (PMC3668281; doi:10.1186/1471-213X-13-16)

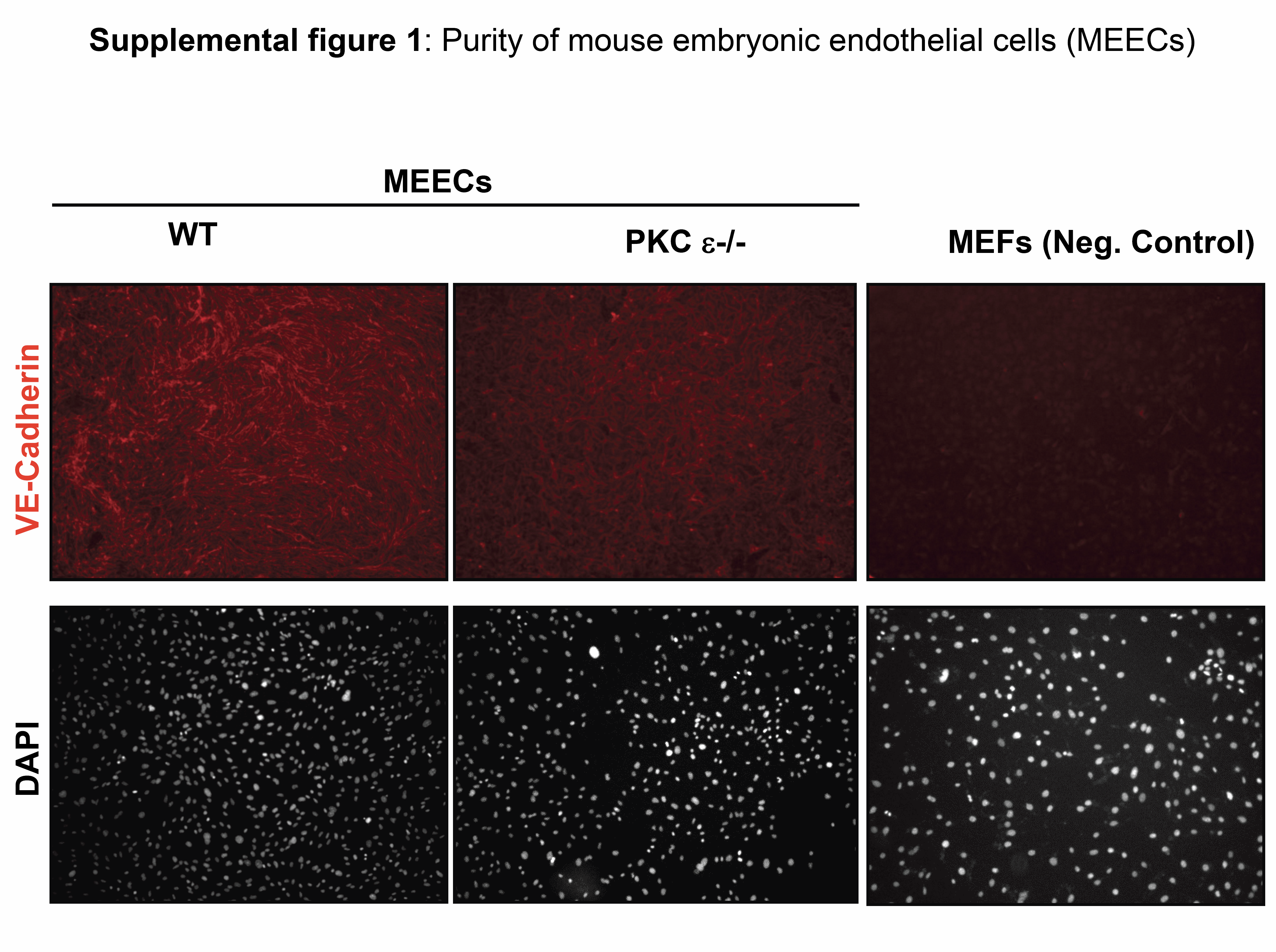

Supplement: Additional file 1: Figure S1 — Purity of MEEC lines. VE-Cadherin was detected via immunofluorescence microscopy to confirm the purity of WT and PKCϵ deficient MEECs. Nearly all cells appeared positively stained in both genotypes. WT mouse embryonic fibroblasts (MEFs) were used as a negative control. [file 1471-213X-13-16-S1.tiff]
